# Targeting Solid Tumors With BTK Inhibitors

**DOI:** 10.3389/fcell.2021.650414

**Published:** 2021-04-14

**Authors:** Fatih M. Uckun, Taracad Venkatachalam

**Affiliations:** Immuno-Oncology Program, Ares Pharmaceuticals, LLC, St. Paul, MN, United States

**Keywords:** BTK-Bruton’s tyrosine kinase, solid tumors, breast cancer, prostate cancer, metastasis

## Abstract

The repurposing of FDA-approved Bruton’s tyrosine kinase (BTK) inhibitors as therapeutic agents for solid tumors may offer renewed hope for chemotherapy-resistant cancer patients. Here we review the emerging evidence regarding the clinical potential of BTK inhibitors in solid tumor therapy. The use of BTK inhibitors may through lead optimization and translational research lead to the development of new and effective combination regimens for metastatic and/or therapy-refractory solid tumor patients.

## Bruton’s Tyrosine Kinase as a Validated Molecular Target in Cancer Cells

**FIGURE 1:**
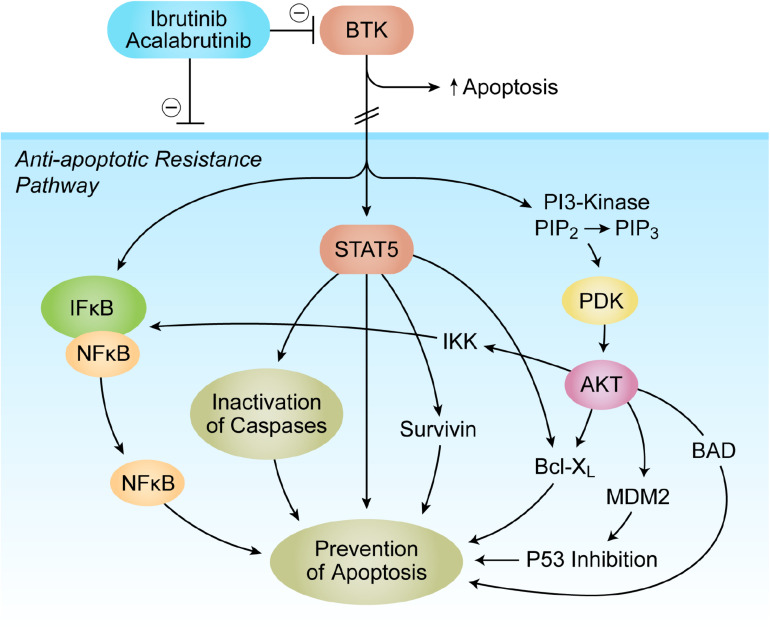
Bruton’s tyrosine kinase (BTK) as a Master Regulator of Apoptosis in tumor microenvironment (TME). The anti-apoptotic BTK-PI3K-AKT signaling pathway is critical for the survival of tumor cells. Multiple antiapoptotic signaling molecules and pathways linked to NF-κB, PI3-K/AKT, and STAT5 are regulated by BTK. See text for a detailed discussion.

Bruton’s tyrosine kinase (BTK) is linked to multiple signal-transduction pathways and networks, regulating survival, activation, proliferation, and differentiation of B-lineage lymphoid cells (Vassilev et al., 1999; Mahajan et al., 2001; Uckun and Sudbeck, 2001; Uckun et al., 2002, 2004, 2007b; Uckun and Malaviya, 2004; Uckun and Qazi, 2010; Burger, 2019). BTK is expressed in neoplastic cells from patients with B-lineage lymphoid malignancies (Vassilev et al., 1999; Mahajan et al., 2001; Uckun and Sudbeck, 2001; Uckun et al., 2002, 2004, 2007b; Uckun and Malaviya, 2004; Uckun and Qazi, 2010; Bond et al., 2019; Burger, 2019; Kim, 2019). The anti-apoptotic BTK-PI3K-AKT signaling pathway is critical for the survival of tumor cells (Figure 1). Multiple antiapoptotic signaling molecules and pathways linked to NF-κB, PI3-K/AKT, and STAT5 are regulated by BTK. Consequently, BTK has emerged as a new molecular target for treatment of B-lineage leukemias and lymphomas as well as —more recently—solid tumors. BTK inhibitors (BTKi) have replaced several chemotherapy-based regimens in standard of care for some of the B-lineage lymphoid malignancies, especially in patients with CLL and mantle cell lymphoma (MCL) (D’Cruz and Uckun, 2013; Thompson and Burger, 2018; Bond et al., 2019; Jurczak et al., 2019; Kim, 2019). The first-generation BTKi ibrutinib binds covalently to a cysteine residue (Cys481) in the active site of the ATP-binding domain of BTK. Second-generation BTKi were designed to have fewer off-target effects than ibrutinib (D’Cruz and Uckun, 2013). The second-generation BTKi acalabrutinib also binds Cys481 in the BTK active site, and it is FDA approved for the treatment of adults with CLL or SLL (Feng et al., 2019). Another second-generation BTKi, zanubrutinib, received an accelerated approval from the FDA for the treatment of adult patients with MCL (Feng et al., 2019). A novel oncogenic isoform of BTK with a survival-promoting function is abundantly expressed in breast cancer, ovarian cancer, prostate cancer, and colorectal cancer (Eifert et al., 2013; Guo et al., 2014; Kokabee et al., 2015; Wang et al., 2016; Conconi et al., 2017; Molina-Cerrillo et al., 2017; Campbell et al., 2018; Chen et al., 2018; Lavitrano et al., 2019). Overexpression of BTK in solid tumor cells was associated with elevated expression of genes with functions related to cell adhesion, cytoskeletal structure, and extracellular matrix as well as aggressiveness of the cancer (Guo et al., 2014). Knockdown of these isoforms by RNA interference using siRNA or treatment with BTKi like ibrutinib resulted in inhibition of growth as well as apoptosis and enhanced chemosensitivity of cancer cells (Eifert et al., 2013; Guo et al., 2014; Kokabee et al., 2015; Wang et al., 2016; Molina-Cerrillo et al., 2017; Campbell et al., 2018; Chen et al., 2018; Lavitrano et al., 2019). Grassilli et al. (2016) reported that this 65-kDa novel isoform of BTK is expressed in colorectal cancer cells in a mitogen-activated protein kinase (MAPK)-dependent manner. Furthermore, BTKi ibrutinib inhibited the proliferation of human colorectal cancer cell lines *in vitro* (Grassilli et al., 2016) and enhanced the chemosensitivity of drug-resistant colorectal cancer cells (Ianzano et al., 2016). Inhibition of BTK also reduced the clonogenicity of cancer stem cells and decreased their resistance to chemotherapy drugs (Metzler et al., 2020). BTKi were shown to synergize with the standard chemotherapy drug 5-fluorouracil against chemotherapy-resistant colorectal cancer cells (Lavitrano et al., 2019). First-generation BTKi LFM-A13 caused apoptosis in human colorectal cancer cells and exhibited potent anticancer activity against xenografted human colorectal cancer cells in mice both as a single agent and in combination with erythropoietin (Tankiewicz-Kwedlo et al.,2018a,b). p65BTK was also detected in non-small cell lung cancer (NSCLC) cell lines, including those with mutant KRAS, and treatment of these cell lines with BTKi resulted in loss of viability and inhibition of clonogenic growth (Giordano et al., 2019). Furthermore, BTKi enhanced the sensitivity of NSCLC cell lines to standard chemotherapy drugs (Giordano et al., 2019). Wei et al. (2016) reported that human glioblastoma (GBM) cells express p77BTK, and downregulation of BTK expression inhibits the antiapoptotic AKT/mTOR pathway, and BTKi ibrutinib exhibits *in vivo* antitumor activity in a mouse xenograft model of GBM. Recently, Sala et al. (2019) reported that p65BTK is expressed in patient-derived human glioma cells, and BTKi diminish their viability.

Both BTK and the related TEC kinases ETK and BMX are abundantly expressed in prostate cancer cells, and knockdown of BTK expression in prostate cancer cells results in reduced proliferative activity (Guo et al., 2014; Kokabee et al., 2015; Chen et al., 2018). Inhibition of BTK and ETK with a small molecule inhibitor caused inhibition of proliferation, clonogenic growth, invasiveness of human prostate cancer cell lines both in *in vitro* and an *in vivo* SCID mouse xenograft model (Guo et al., 2014). BTK inhibition was also associated with substantial downregulation of oncogenic genes, such as MYC, in prostate cancer cell lines and enhances their chemosensitivity to standard drugs such as docetaxel (Guo et al., 2014). Likewise, ovarian cancer cells express BTK, and high expression levels are correlated with aggressiveness of disease, progression to Stage IV metastatic cancer, and poor survival (Zucha et al., 2015).

Similarly, numerous studies have shown that BTK inhibition causes substantial cytotoxicity to HER2^+^ breast cancer cells, inhibits their proliferation and clonogenicity, and diminishes their resistance to chemotherapy both *in vitro* and *in vivo* (Eifert et al., 2013; Chen et al., 2016; Wang et al., 2016; Metzler et al., 2020; Wen et al., 2020). The results obtained with non-specific BTKi like ibrutinib should be interpreted with due caution because several other kinases, including ERBB2/HER-2 that have ibrutinib-binding cysteine residues in their kinase domains are inhibited by ibrutinib (Berglof et al., 2015). Nonetheless, LFM-A13, a first-generation BTKi with no HER-2 or EGF-R inhibitory activity, also exhibited antitumor activity in the MMTV/neu transgenic mouse model of HER2-positive breast cancer. It was at least as effective as the standard breast cancer drugs paclitaxel and gemcitabine, and it improved the efficacy of paclitaxel (Uckun, 2007; Uckun et al., 2007a). In the DMBA breast cancer model, the BTKi LFM-A13 significantly delayed spontaneous tumor appearance as well as tumor progression, and it substantially improved tumor-free survival (Güven et al., 2020). The tumors developing despite chemoprevention with LFM-A13 were small and grew slowly. Hence, BTK inhibition prevented the development of aggressive and rapidly progressive mammary gland tumors.

Bruton’s tyrosine kinase inhibition is also associated with inhibition of tumor growth in pancreas cancer (Massó-Vallés et al., 2015; Gunderson et al., 2016). In view of the broad-spectrum anti-cancer activity exerted by BTKi in various non-clinical cancer models, BTK inhibition with ibrutinib and acalabrutinib has been evaluated in several proof-of-concept solid tumor trials (e.g., NCT02403271, NCT03525925, NCT03379428, NCT02599824, and NCT02562898) aimed at assessing its potential clinical benefit in patients with solid tumors, including ovarian cancer, breast cancer, lung cancer, prostate cancer, and pancreas cancer (Massó-Vallés et al., 2016; Hong et al., 2019; Overman et al., 2020). The maturation of data from these trials will provide valuable insights regarding the clinical impact potential of BTK inhibition as part of multimodality treatment regimens for difficult-to-treat forms of cancer. The reported suppression of cancer stemness in non-clinical models awaits confirmation from clinical proof-of-concept studies (Pan et al., 2020).

## Bruton’s Tyrosine Kinase and Tumor Microenvironment

**FIGURE 2:**
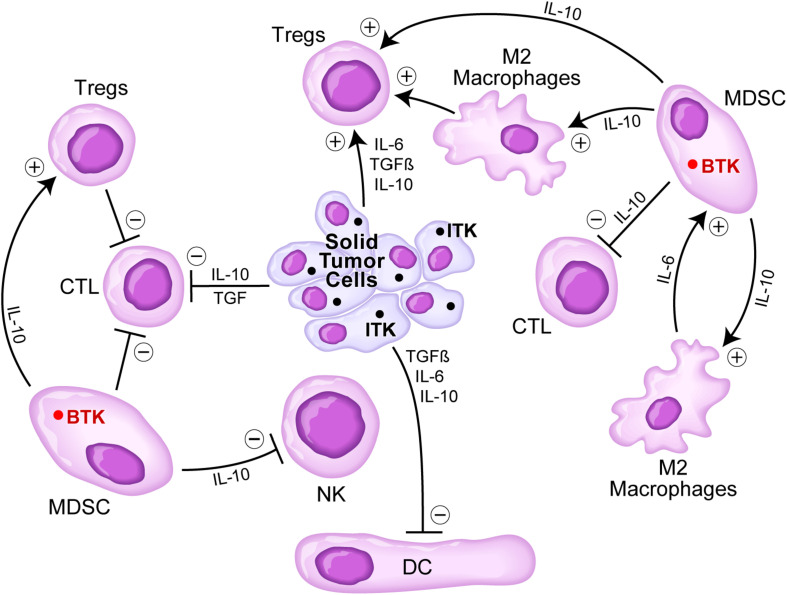
Immunosuppressive TME in solid tumors. Tumor cells secrete several cytokines including IL-6, TGFβ, and IL-10 that inhibit DCs, CTLs, but stimulate regulatory T cells (Tregs). Myeloid-derived suppressor cells (MDSCs) are stimulated via IL-6 by M2 macrophages and stimulate M2 macrophages as well as Tregs via IL-10, but they inhibit via IL10 CTLs and NK cells. Since solid tumor cells express the TEC kinase inducible T-cell kinase (ITK) related to BTK and MDSCs express BTK, BTK inhibition might offer an effective strategy to overcome an immunosuppressive TME. See text for a detailed discussion.

Several cellular elements of the tumor microenvironment (TME) of solid tumor patients contribute to the immune evasion, proliferation, and drug resistance of tumor cells, including myeloid-derived suppressor cells (MDSCs), tumor-associated M2-like, “alternatively activated,” macrophages, and regulatory T cells (Tregs) (Figure 2). Notably, some solid tumors abundantly express IL-2 inducible T-cell kinase (ITK), a TEC kinase related to BTK (Figure 2). It has been reported that ITK inhibition by the existing BTKi can result in improved T-cell responses via reduced production of IL-10 and TGFβ that have immunosuppressive effects (Dubovsky et al., 2013; Sagiv-Barfi et al., 2015; Chen et al., 2016; Stiff et al., 2016). Furthermore, in a breast cancer mouse model, BTKi ibrutinib improved the efficacy of anti-PD-L1 treatment (Sagiv-Barfi et al., 2015). On the other hand, BTK inhibition may potentially reduce the potency of immune checkpoint inhibitors. That is because BTK expressing tumor infiltrating cells within the TME include memory B cells that cooperate with memory T-cells to ensure a robust immune response to cancer cells. A recent study in which >500 lung adenocarcinoma cases were analyzed for possible contribution of BTK to an immune-dominant profile of the TME revealed that BTK expression in the TME was associated with a less aggressive disease and an improved survival outcome (Bi et al., 2020). MDSCs in the TME have been shown to express BTK (Figure 2), and it has been proposed that BTK inhibition may therefore lift the MDSC-mediated suppression of the antitumor immunity within the TME (Stiff et al., 2016). The potential effects of BTK inhibition on the tumor microenvironment and the potency of immune-checkpoint inhibitors will be clarified in part by the ongoing clinical trials that combine BTK inhibition with immune checkpoint blockade.

## Coumarins as a New Class of Bruton’s Tyrosine Kinase Inhibitors

Coumarins are derivatives of 2H-1-benzopyran-2-one, which naturally occurs in plants as free coumarins or their glycoside derivatives (Kashman et al., 1992; Currens et al., 1996; McKee et al., 1998; Creagh et al., 2001; Shokoohinia et al., 2018; Bhatia and Rawal, 2019; Kawai et al., 2019; Li et al., 2019; Lin et al., 2019; Makowska et al., 2019; Ramdani et al., 2019; Selvaraj et al., 2019; Wang et al., 2019; Zhang and Xu, 2019). Coumarins have been known for their proapoptotic anticancer activity with inhibitory effects on tumor-promoting signal transduction pathways as well as antiviral activity (Kashman et al., 1992; Currens et al., 1996; McKee et al., 1998; Creagh et al., 2001; Shokoohinia et al., 2018; Bhatia and Rawal, 2019; Kawai et al., 2019; Li et al., 2019; Lin et al., 2019; Makowska et al., 2019; Ramdani et al., 2019; Selvaraj et al., 2019; Wang et al., 2019; Zhang and Xu, 2019). The naturally occurring coumarin derivatives, (+)-calanolide A and (–)-calanolide B, have been identified as inhibitors of non-nucleoside HIV-1-specific reverse-transcriptase inhibitory activity (Kashman et al., 1992; Currens et al., 1996; Creagh et al., 2001). In recent years, the coumarin scaffold has also been used in developing anticancer drugs. Several semi-synthetic calanolide derivatives have been developed as antiviral drug candidates (Creagh et al., 2001; Sagiv-Barfi et al., 2015; Chen et al., 2016). Researchers have hybridized coumarin moieties with other anticancer pharmacophores as a strategy of developing novel anticancer drugs (Flavin et al., 1996; Bhatia and Rawal, 2019; Kawai et al., 2019; Lin et al., 2019; Makowska et al., 2019; Ramdani et al., 2019; Selvaraj et al., 2019; Wang et al., 2019; Zhang and Xu, 2019). In addition, some natural coumarins such as Psoralidin (Li et al., 2019) and Osthol (Shokoohinia et al., 2018) have been reported to exhibit potent *in vitro* and *in vivo* anticancer activity. Coumarin–fatty acid conjugates as well as coumarin hybrids generated via coupling with isoxazole, thiazole, monastrol, chalcone, triazole, sulfonamide, triphenylethylene, benzimidazole, pyran, imidazole, stilbene, estrogen, or phenylsulfonylfuroxan exhibited promising pro-apoptotic anticancer activity (Bhatia and Rawal, 2019; Kawai et al., 2019; Makowska et al., 2019; Selvaraj et al., 2019; Zhang and Xu, 2019).

We discovered that the crystal structure of the BTK kinase domain reveals a distinct 7 Å × 7 Å rectangular binding pocket near the hinge region of the BTK kinase domain with Leu-460, Tyr-476, Arg-525, and Asp-539 residues occupying the corners of the rectangle (Uckun and Sudbeck, 2001; Uckun et al., 2002, 2004; Uckun and Malaviya, 2004). The overall geometry inside the active site near the hinge region was estimated to be sufficient to accommodate the rationally designed BTK-inhibitory calanolide derivatives (Uckun and Sudbeck, 2001; Uckun et al., 2002, 2004; Uckun and Malaviya, 2004).

## Conclusion

The repurposing of FDA-approved BTKi as therapeutic agents for solid tumors may offer renewed hope for chemotherapy-resistant cancer patients. Advanced prostate cancer has a dismal outcome, and patients with metastatic disease are in urgent need for therapeutic innovations (Litwin and Tan, 2017; Siegel et al., 2019). Androgen deprivation by both chemical and surgical castration is initially useful in the treatment of metastatic prostate cancer, but patients ultimately enter the castration-resistant stage (CRPC) where there is no effective treatment (Litwin and Tan, 2017; Siegel et al., 2019). Likewise, advanced and metastatic breast cancer patients, especially those with triple-negative breast cancer (TNBC) are in urgent need for therapeutic innovations (Bergin and Loi, 2019; Pandy et al., 2019; Thill et al., 2019). The discovery of effective treatment strategies using chemotherapy drugs, precision medicines, biologics, and natural compounds is a major area of translational research emphasis in contemporary oncology, especially for breast cancer and prostate cancer. Re-purposed BTKi currently approved for B-lineage lymphoid malignancies as well as new BTKi with enhanced potency against solid tumors may provide the basis for more effective combination regimens.

MDSCs in the TME have been shown to express BTK, and it has been proposed that BTK inhibition may therefore lift the MDSC-mediated suppression of the antitumor immunity within the TME (Stiff et al., 2016). However, BTK inhibition may potentially reduce the potency of immune checkpoint inhibitors because of disruption of cognate interactions between BTK expressing memory B-cells and memory T-cells. Whether or not BTK inhibition will result in a clinically meaningful inhibition of MDSC and/or help overcome resistance to ICI awaits clinical proof of concept. A recent randomized study in metastatic urothelial cancer patients that evaluated a combination of the ICI pembrolizumab with acalabrutinib failed to show any benefit from this combination vs. pembrolizumab alone (Zhang et al., 2020). On the other hand, a promising efficacy signal was obtained during the interim analysis of a randomized study (Clinicaltrials.gov identifier: NCT02599324) employing ibrutinib plus paclitaxel in patients with metastatic urothelial carcinoma (Castellano et al., 2019). Likewise, a combination of ibrutinib with the anti-EGF receptor antibody cetuximab showed moderate activity in patients with metastatic colorectal cancer (Oh et al., 2020). The identification of the most effective and best-tolerated combination regimens will likely require rationally designed clinical studies with multiple treatment cohorts enrolling in parallel and adaptive trial designs.

## Author Contributions

FU and TV have made significant and substantive contributions to the study, reviewed and revised the manuscript, and provided final approval for submission of the final version. FU conceived the study, designed the evaluations reported in this manuscript, directed the data compilation and analysis, analyzed relevant data, and prepared the initial draft of the manuscript. Both authors contributed to the article and approved the submitted version.

## Conflict of Interest

FU was an employee of Ares Pharmaceuticals, LLC. TV was a consultant of Ares Pharmaceuticals, LLC.
